# Has COVID-19 Been the Great Leveler? The Changing Use of Intergenerational Digital Communications Amongst Older People in England During the Pandemic

**DOI:** 10.1177/07334648231153385

**Published:** 2023-01-20

**Authors:** Athina Vlachantoni, Jane Falkingham, Maria Evandrou, Min Qin

**Affiliations:** 1 ESRC Centre for Population Change (Connecting Generations), Faculty of Social Sciences, 7423 University of Southampton, Southampton, UK; 2 Centre for Research on Ageing, Faculty of Social Sciences, 7423 University of Southampton, Southampton, UK

**Keywords:** digital communication, disparity, internet experiences, intergenerational solidarity, social distancing, COVID-19 pandemic

## Abstract

This research examines patterns of intergenerational digital contact before and during the COVID-19 pandemic in England, using data from the English Longitudinal Study of Ageing (ELSA) Wave nine and the first Wave of the ELSA COVID-19 Sub-study. Multivariate binary logistic regressions were applied to assess the determinants of frequent intergenerational digital communication. The findings indicate that when the pandemic began, many older persons shifted towards more frequent intergenerational digital contact, but a small minority shifted away. As a result, the pre-existing gender gap amongst older people in the use of digital communication technology narrowed, as did the disparity associated with family relationship closeness. However, pre-pandemic gaps in the intergenerational digital connection between internet users and non-users widened during the pandemic. Overall, the results suggest that the pandemic resulted in more frequent digitally-mediated social interactions within the family, which may strengthen ties between older and younger family members.

**What this paper adds**

• During the pandemic, many older persons shifted towards frequent intergenerational digital contact. Such shifts were observed across all sub-groups with different demographic, socio-economic characteristics and internet skills.
• The gender gap and the discrepancy related to the closeness of family relationships in digital communication narrowed. However, inequalities in the intergenerational digital connection between internet users and non-users widened.
• Intergenerational digital communication played a role in meeting older people’s health and social care needs and mitigating the negative impact on well-being.

**Applications of study findings**

• Thanks to the increased uptake of information and communication technologies (ICT), many older persons experienced greater associational solidarity with their families during the COVID-19 pandemic. Older adults will utilise ICT more frequently in the coming years to assist them with daily tasks or to preserve or grow their social networks.
• There remains a sub-group of older people who risk being left behind. To bridge the remaining digital divide, education programs for digital skills will need to highlight the benefits of being online to overcome any apprehension about engagement. The design of ICT will need to respond to older adults' needs and capabilities.

## Introduction

Research on family and kinship shows that family ties remain strong in England (Evandrou et al., 2018; Grundy & Shelton, 2001), similarly to other developed nations (Silverstein et al., 2010), despite societies generally becoming more affluent and welfare systems maturing and becoming more comprehensive. Most mid-life adults living in England reported having received some support, such as finances, accommodation and childcare from their parents earlier in life; and many of them now provide care, such as transportation, shopping, financial assistance, dressing and bathing, to their older parents (Evandrou et al., 2018). Parents and their offspring usually maintain contact, feel a sense of obligation toward one another and continue to exchange instrumental, financial and emotional support across the life course (Suitor et al., 2015).

COVID-19 had direct and indirect health impacts in England. It resulted in more severe morbidity and greater mortality among older adults than in other age groups. During the pandemic, outside of admissions for COVID-19 itself, a significant reduction in hospital activity was recorded both for inpatient admissions and outpatient visits. People living in the community with long-term care needs also saw formal social support service access and usage reduced significantly (Department of Health and Social Care (DHSC) & Office for National Statistics (ONS), 2021). The pandemic disrupted well-established family interactions, such as normal modes of communication and emotional and practical support (Derrer-Merk, et al., 2022). Family members had to communicate by phone or video conferencing and always keep a physical distance. Support was given at a distance rather than face-to-face. Grandparents were no longer providing support for grandchildren, but adult children were providing more support to parents (e.g. shopping). Those who followed the shielding advice or chose to separate themselves experienced the stress of not being physically close to loved ones. This decision impacted their well-being, and many older people reported feeling isolated and lonely (Ibid).

The intergenerational solidarity model (Bengtson & Roberts, 1991; Silverstein & Bengtson, 1997) proposes six interrelated components of family solidarity: affectional (emotional closeness), associational (frequency of contact), normative (norms of obligation), consensus (agreement about values), structural (geographical proximity) and functional (exchange of support). Recently, the concept of associational solidarity has been extended to include ‘digital solidarity’ to consider the role of technology in maintaining contact between generations (Peng et al., 2018). Digital communication has been argued to enhance intergenerational cohesion beyond traditional modes of communication, such as face-to-face visits that demand more time and coordination (Rafnsson et al., 2022). It is essential to exchange affectional and instrumental support in the face of economic or health emergencies (Webb & Dickson, 2012). Digital technologies may be especially valuable for older persons facing barriers to travel due to health limitations, transportation costs and social isolation, facilitating regular contact with their adult children and other family members (Freeman et al., 2020). In a recent review paper on ageing families, Fingerman et al., (2020) highlighted the implications of technological advances for intergenerational communication, illustrating how digital communication had enabled and strengthened the interdependence between older and younger generations. Recent research found that older parents with distant but digitally connected relationships with their adult children reported better mental health than those with detached and ambivalent relationships during the COVID-19 pandemic (Hwang et al., 2022), providing further support for intergenerational digital communication being included as digital solidarity in the intergenerational solidarity paradigm.

## Research Objectives and Questions

This study aims to explore the patterns of digital communication use among older adults to connect with their families before and during the COVID-19 pandemic. Intergenerational digital communication includes emailing, texting and video calling to family members (parents, children, grandchildren and siblings) outside the household. The study addresses the following research questions:

RQ1 How were socio-demographic factors and internet experiences associated with intergenerational digital communication *before* the COVID-19 pandemic?

RQ2 How did intergenerational digital communication change during the pandemic? Who shifted towards, and who shifted away, from frequent intergenerational digital communication during the early phase of the pandemic?

RQ3 To what extent have the changes experienced during the COVID-19 crisis reinforced or mitigated existing inequalities in the use of digital communications and intergenerational connectedness?

The use of technology in fostering social relationships through the internet has grown quickly in the first two decades of the twenty-first century with an increase in communication and meaningful social interactions between peers and family that bridge distance and generations (Reis et al., 2021). In Great Britain, in January–February 2020, 96% of households had internet access. The proportion of households with one adult aged 65 years and over with an internet connection increased by seven percentage points since 2019 to 80% (ONS, 2020). Older adults who use the internet are most likely to do so as a means of communication (Age UK, 2018). However, despite the expansion of connectivity, digital inequalities have persisted and over time may be argued to have deepened with a sub-group of the older population at risk of being left behind (Blank et al., 2020; ONS, 2019).

The COVID-19 pandemic and the associated physical distancing measures have resulted in social isolation and loneliness among older people being more prevalent (Holt-Lunstad, 2021; Vlachantoni et al., 2022). Older adults have been identified as being at a higher risk of poor health outcomes if infected with the coronavirus and, in many countries, have been subjected to greater restrictions regarding physical contact with others (Dahlberg, 2021). Given the need to reduce physical contact during the COVID-19 pandemic, family members often turned to technology to maintain contact (Glazer, 2020; Nguyen et al., 2020). A recent empirical study found that more frequent internet use, particularly for communication during the pandemic, was associated with enhanced quality of life in older adults (Wallinheimo & Evans, 2021). Regular use of digital technology reduced feelings of loneliness, anger/irritability and boredom, and increased feelings of belonging via the perception of social support (Gabbiadini et al., 2020). Relatively little is known, however, about the characteristics of those older people who shifted towards or away from digital forms of communication during the early stages of the pandemic, and how such changes have affected pre-existing digital disparities and intergenerational connectedness. Improved understanding of the patterns of use, and importantly non-use, of digital communication technology will provide vital evidence to inform the design of interventions to enhance family solidarity and counter isolation in later life, providing improved opportunities for meaningful contact for those older people who want it.

## Factors Influencing Intergenerational Digital Communication

Previous research has shown that demographic characteristics and socio-emotional factors are associated with both family associational solidarity and intergenerational digital communication (ONS, 2020; Peng et al., 2018; Suitor et al., 2015). Increasing age has been found to reduce the likelihood of internet use and digital communication (Elliot et al., 2014), although it has been argued that chronological age does not affect communication technology use beyond the effects of cohort membership (Peng et al., 2018). Thus, it is not age per se that may be important, but rather one’s age at a particular moment in time, with those generations born in the earlier part of the twentieth century being most at risk of digital exclusion (Ibid).

Gender has also been found to play a role in intergenerational digital communication. Previous research has shown that men tend to use the internet more for informational purposes whilst women are more likely to use it for social and expressive purposes (Jackson et al., 2001; Kimbrough et al., 2013). However, regarding communication during the COVID-19 pandemic, a recent study in the Netherlands found men to be more engaged digitally than women (van Deursen, 2020). Whether such gender differences apply across national contexts during pandemic conditions remains unclear.

At the same time, prior research has highlighted socio-economic differentials, with better-educated persons being more likely to use communication technology with their offspring than their less-educated counterparts (Peng et al., 2018). Education leads to greater openness, change and supportive learning environments that motivate technology adoption among older adults (Hill et al., 2008). In the COVID-19 pandemic, individuals with greater economic resources were shown to use the internet more efficaciously and productively (Nguyen et al., 2021). By contrast, individuals with more social resources are more likely to have access to family, friends or other contacts on the internet, although research has shown that parents may favour particular children in terms of emotional closeness and support (Suitor et al., 2007). Moreover, individuals need to possess adequate skills to reap the benefits of digital media use; those with the skills who are already ‘digital privileged’ are more likely to use the information and communication opportunities provided by the internet to inform themselves and connect with others about the COVID-19 pandemic (Nguyen et al., 2021, van Deursen, 2020). The broader literature on technology use has shown that older adults in worse health are less likely to use communication technology (Elliot et al., 2014; Gell et al., 2015). Poor or deteriorating health may deter people from learning about communication technology in the first place or induce older users to discontinue using it (Ibid).

During the COVID-19 pandemic, digital experiences and habits are likely to be modified, especially during periods of ‘lockdown’ and widespread physical distancing. Since many individuals may be cut-off from regular social interaction, they may opt for voice and video calls as the next best alternative (Ipsos, 2020; Wen, 2020). One study observed a substantial increase in digital communication among US adults, including voice calls, social media, video calls, emailing and playing online games during the early phase of the pandemic (Nguyen et al., 2020). Several cross-sectional studies conducted during the pandemic have highlighted that people with greater socio-economic and digital resources were more likely to increase their digital communication (Nguyen et al., 2021, van Deursen, 2020), pointing towards the COVID-19 crisis reinforcing existing inequalities. However, given their cross-sectional design, these studies are limited in reaching any definitive conclusions. The present study, by adopting a longitudinal design, aims to add to the literature and shed new light on the extent to which digital inequalities are amplified or mitigated in times of crisis, distinguishing between different modes of intergenerational digital communication.

Given the empirical and survey data discussed above, we expect that older adults with greater socio-economic and digital privilege will be more likely to engage in intergenerational digital communication prior to the pandemic. During the COVID-19 pandemic, we anticipate that some older adults who had not previously engaged in frequent intergenerational digital communication will have increased their use of such technology, although we also expect that there will be a group of older adults who will have shifted away from frequent digital contact with families under the constraints of the pandemic. Given the increased physical distancing and the associated adoption of digital communication technology by new users, we hypothesise that pre-existing digital disparities will have narrowed during the pandemic, and as a result intergenerational ties between older and younger family members may have strengthened. However, there may also be a group of older people who have continued to be digitally excluded; understanding this group is critical for informing the design of future policy in the area of digital and social inclusion.

## Data and Methods

This study uses data from the English Longitudinal Study of Ageing (ELSA) cohort study (Steptoe et al., 2013). ELSA collects data from a national representative sample of adults aged 50 years and over in England every 2 years. In June/July 2020, ELSA conducted a COVID-19 sub-study (Steptoe et al., 2021) to investigate the effects of the COVID-19 pandemic on middle-aged and older persons in England. The data collection of the sub-study combined internet and telephone assessments. The final response rate of the first Wave of the ELSA COVID-19 Sub-study was 75% (7040 completed interviews from a sample of 9392 study respondents), with 83% of the surveys completed online and 17% on the phone. The analysis here uses linked data from ELSA Wave 9 (Banks et al., 2021) conducted from June 2018 to May 2019, providing the pre-pandemic ‘baseline’, and the COVID-19 sub-study, providing an insight into the circumstances of older people during the first phase of the pandemic. The institutional review board approved ELSA Wave 9 and the COVID-19 study.

The analytical sample used here included all respondents aged 65 and over currently living in a private household and having at least one surviving immediate family member living in another household, including adult children, parents, grandchildren or siblings. The total eligible sample in Wave 9 was 5048. Amongst these, 4180 respondents were followed up in the COVID-19 sub-study, with 868 respondents lost to follow-up, accounting for 17.2% of the total. Compared with the baseline study sample, those respondents lost to follow-up were more likely to be older and in a less privileged socio-economic position. They also reported poorer health and more difficulties in carrying out Activities of Daily Living (ADLs). Moreover, they were more likely never to use the internet and have no frequent contact with a family member, or to have only non-digital contact with family members. There were no differences by gender or whether they had a close family member (Supplementary Table 1). To address issues of sample attrition and non-response bias, longitudinal weights were applied for individual-level changes between ELSA Wave 9 and the Covid-19 study. As all participants for the COVID-19 sub-study were selected from the existing ELSA sample, these longitudinal weights calculated by the survey team were specifically designed for analysis of individual-level change between ELSA wave 9 and wave 1 of the COVID-19 study, being the product of a (trimmed) non-response weight and the wave 9 cross-sectional weight. (The non-response weight adjusts for non-response to wave 1 of the COVID-19 study, contingent on response to ELSA wave 9.) (Steptoe et al., 2021). The dependent variables were derived from the ELSA Wave 9 and the COVID-19 sub-study, with the predictive variables drawn from the ELSA Wave 9.

### Measures: Dependent Variables

The ELSA Wave 9 questionnaire included information on the frequency and mode of interaction by study respondents with their children and family. A typical question asked:

*‘How often, on average, do you do each of the following (meet up/speak on the phone/write or email/send or receive text messages) with any of your (children/family), not counting any who live with you?’*

Answers to each question were originally recorded on a 6-item ordinal Likert scale, with response options ranging from ‘less than once a year or never’ to ‘three or more times a week’.

In the ELSA COVID-19 Sub-study, all respondents were asked:

*‘In the past month, how often have you done the following (speaking on the phone/video-calling e.g., Skype, FaceTime, etc./write or email/send or receive text messages) with any of your immediate family (parents, children, grandchildren and brothers and sisters), not counting any who live with you?’*

Answers to each question were originally recorded on a 4-item ordinal Likert scale, with response options ranging from ‘less than once a week or never’ to ‘daily’.

This study defines digital communication as emailing, texting or video-calling contact. Intergenerational digital communication was digital contact with any children or family members outside the household. We distinguish between ‘frequent’ and ‘less frequent’ contact using a threshold of ‘at least once a week’.

The first dependent variable ‘frequent intergenerational digital communication before the COVID-19 pandemic’, was coded as a binary variable (1 if they had frequent intergenerational digital contact vs. 0 for other categories).

Another two binary dependent variables were derived to capture change over time, that is, ‘shift towards frequent intergenerational digital communication’ and ‘shift away from frequent intergenerational digital communication’, measuring the changes before and during the pandemic. First, a variable to measure ‘frequent intergenerational digital communication during the COVID-19 pandemic’ was created using the same binary cut-off as that used prior to the pandemic. The ‘shift towards frequent intergenerational digital communication’ was then created as a binary variable (1 if respondents changed from non-frequent intergenerational digital contact pre-pandemic to frequent intergenerational digital contact during the pandemic vs. 0 for other categories). Similarly, the ‘shift away from frequent digital contact’ was coded as 1 if respondents changed from frequent intergenerational digital communication pre-pandemic to non-frequent digital contact during the pandemic versus 0 for other categories.

### Measures: Independent Variables

Independent variables included demographic and socio-economic factors (birth cohort, gender, educational qualification and household wealth), health factors (long-standing illness, the number of Activities of Daily Living (ADL) difficulties, and the number of Instrumental Activities of Daily Living (IADLs) difficulties), familial factors (closeness with family members and living arrangements) and the digital experience (pre-pandemic Internet use). Detailed measurement definitions and operationalisations are presented in the Supplementary Text.

### Analysis Plan

Multivariate binary logistic regressions were applied to assess the determinants (demographic, socio-economic and digital experiences) of frequent intergenerational digital communication before the pandemic and the shifts in frequent intergenerational digital communication during the pandemic. For each binary logistic regression model, the model diagnostics statistics, including checking for specification error (the linktest), multicollinearity (variance inflation factor (VIF)), influential outliers (the deviance residual and the leverage (the hat value)) and goodness-of-fit (likelihood ratio test), were monitored and satisfied.

## Results

### Sample Characteristics

The average age of respondents was 74.9 years (SD = 7.1), 54% were female and 35% had completed A-level or above education. About 60% of respondents reported a long-standing illness, while 18% reported difficulty in performing one or more ADLs. About 29% of respondents lived alone and 6% reported no close family member. Finally, 77% of respondents had used the internet prior to the pandemic.

### How were Socio-Demographic Factors and Internet Experiences Associated with Frequent Intergenerational Digital Communication Pre-Pandemic?

About 54% of respondents reported frequent digital contact with their family members prior to the COVID-19 pandemic (Table 1). There was a gap in frequent intergenerational digital communication by gender and between different birth cohorts, socio-economic position, health, family relationship and internet use (Table 1).

**Table 1.** Descriptive Statistics Regarding Pre-Pandemic Frequent Intergenerational Digital Communication and Shifts in Communication During the Pandemic.

	% Frequent intergenerational digital communication pre-pandemic	*p* value(a)	% Shift towards frequent intergenerational digital communication during the pandemic	*p* value(b)	% Shift away from frequent intergenerational digital communication during the pandemic	*p* value(c)	Disparity changes during the pandemic (percentage points)	Sample % (n)
Total % (n)	54.4 (2392)		25.1 (1052)		4.1 (139)			100.0 (4180)
*Mean Age (SD)*								74.9 (7.1)
*Gender*		<.001		<.001		.088	Narrowed (7.6)	
Men	46.4		30.0		4.8			45.7 (1887)
Women	61.2		21.0		3.5			54.3 (2293)
*Birth cohort*		<.001		.457		<.001	Enlarged	
1946–55	62.7		24.3		2.6		(2.5)	55.6 (2518)
1939–45	51.2		25.5		4.6			23.1 (997)
1930–38	36.4		26.8		7.7			21.4 (665)
*Education*		<.001		.316		.014	Enlarged	
Lower than O-level	46.6		24.7		5.6		(4.4)	36.9 (1149)
O-level	58.9		23.9		3.3			28.6 (1261)
A-level and above	59.1		26.8		3.3			34.5 (1770)
*Wealth quintile*		<.001		.177		.046	Narrowed	
The lowest	47.5		26.9		6.8		(0.1)	15.9 (463)
The second	52.0		23.5		3.6			16.7 (646)
The third	52.3		23.6		3.5			22.7 (994)
The fourth	54.2		28.3		4.2			22.4 (1037)
The highest	63.7		23.3		3.4			22.2 (1040)
*Long-standing illness*		<.001		.200		.089	Narrowed	
No	59.9		23.8		3.3		(0.9)	40.5 (1755)
Yes	50.8		26.0		4.7			59.5 (2425)
*Number of ADL difficulties*		<.001		.015		<.001	Narrowed	
0	56.7		24.2		3.4		(3.2)	81.8 (3558)
1	48.1		25.8		6.5			9.8 (356)
2+	39.7		33.0		8.9			8.4 (266)
*Number of IADL difficulties*		<.001		.455		.001	Enlarged	
0	57.7		24.6		3.5		(2.0)	79.3 (3466)
1	44.8		27.3		5.6			10.7 (407)
2+	39.2		27.1		7.9			10.0 (307)
*Living arrangements*		.001		.385		.791	Narrowed	
Living alone	49.5		26.2		4.3		(1.2)	28 9 (1108)
Living with someone	56.4		24.7		4.1			71.1 (3072)
*Has at least one close family member*		<.001		<.001		.186	Narrowed (19.7)	
No	16.2		41.5		2.0			5.6 (228)
Yes	56.7		24.2		4.3			94.4 (3952)
*Pre-pandemic internet use*		<.001		.088		.057	Enlarged	
Ever use	62.2		25.9		3.7		(5.2)	77.4 (3467)
Never use	27.8		22.5		5.5			22.6 (713)

% was weighted by longitudinal weight (COV19LWGT). Number of respondents was non-weighted. SD: Standard Deviation.

*p*-values (a) compare the binary outcome ‘frequent intergenerational digital communication pre-pandemic’ versus others.

*p*-values (b) compare the binary outcome ‘shift towards frequent intergenerational digital communication due to the pandemic’ versus others.

*p*-values (c) compare the binary outcome ‘shift away from frequent intergenerational digital communication due to the pandemic’ versus others. All *p* values were from Chi-squared tests. Disparity changes due to the pandemic: calculated by the largest net changes (% of shift towards minus % shift away) of each pair of sub-groups within each variable.

Source: Authors’ own analysis of ELSA Wave 9 (2018–19) and COVID-19 sub-study (June/July 2020).

Table 2 presents the results of the multivariate logistic regressions. Model 1 shows the effect of demographic, socio-economic factors and health on the respondents’ frequent intergenerational digital contact pre-pandemic. Being female, of a younger cohort, relatively well educated and wealthier were associated with a higher likelihood of using digital means for communication. By contrast, having a long-standing illness, or more than one ADL or one IADL difficulty were associated with a lower likelihood of frequent use of digital communication. Model 2 included familial factors in the analysis. Those older people who were living with someone or who have close family members had a higher likelihood of digital contact. Model 3 included pre-pandemic internet use. Not surprisingly, older people who never used the internet prior to the pandemic had a much lower likelihood of digital communication. The effect of education and household wealth on frequent intergenerational contact was not significant once pre-pandemic internet use was included in Model 3.

**Table 2.** Logistic Regression Models: Frequent Intergenerational Digital Communication Pre-Pandemic.

	Model1 b (SE)	Model2 b (SE)	Model3 b (SE)
*Birth cohort*			
1946–55 (ref)			
1939–45	−0.44*** (0.08)	−0.45*** (0.08)	−0.33*** (0.08)
1930–38	−0.97*** (0.09)	−0.98*** (0.10)	−0.70*** (0.10)
*Gender*			
Men (ref)			
Women	0.77*** (0.07)	0.76*** (0.07)	0.80*** (0.07)
*Education*			
Lower than O-level (ref)			
O-level	0.27** (0.09)	0.28** (0.09)	0.08 (0.09)
A-level and above	0.36*** (0.09)	0.37*** (0.09)	0.11 (0.09)
*Wealth quintile*			
The lowest (ref)			
The second	0.13 (0.13)	0.11 (0.13)	0.10 (0.14)
The third	0.11 (0.12)	0.05 (0.12)	−0.04 (0.13)
The fourth	0.11 (0.12)	0.05 (0.13)	−0.12 (0.13)
The highest	0.38** (0.12)	0.34* (0.13)	0.11 (0.14)
*Long-standing illness*			
No (ref)			
Yes	−0.18** (0.07)	−0.19** (0.07)	−0.19** (0.07)
*Number of ADL difficulties*			
0 (ref)			
1	−0.01 (0.12)	0.03 (0.13)	0.04 (0.13)
2+	−0.31ǂ (0.16)	−0.31^ǂ^ (0.17)	−0.40* (0.17)
*Number of IADL difficultie*			
0 (ref)			
1	−0.32** (0.12)	−0.30* (0.12)	−0.32** (0.12)
2+	−0.25 (0.15)	−0.21 (0.16)	−0.04 (0.16)
*Living arrangements*			
Alone (ref)			
With someone		0.16* (0.08)	0.19* (0.08)
*Has at least one close family member*			
No (ref)			
Yes		2.21*** (0.20)	2.22*** (0.20)
*Pre-pandemic internet use*			
Ever use (ref)			
Never use			0.27*** (0.10)
N	4180	4180	4180
LR chi2	363.07	550.14	719.28
Prob > chi2	<0.001	<0.001	<0.001
Pseudo R2	0.0636	0.0964	0.1260

^ǂ^*p* < .1. *p < .05. **p < .01. ***p < .001.

Source: Authors’ own analysis of the ELSA Wave 9 (2018–19) and COVID-19 sub-study (June/July 2020).

### Who Shifts Towards and Who Shifts Away from Frequent Intergenerational Digital Communication During the Pandemic?

**Figure 1.**
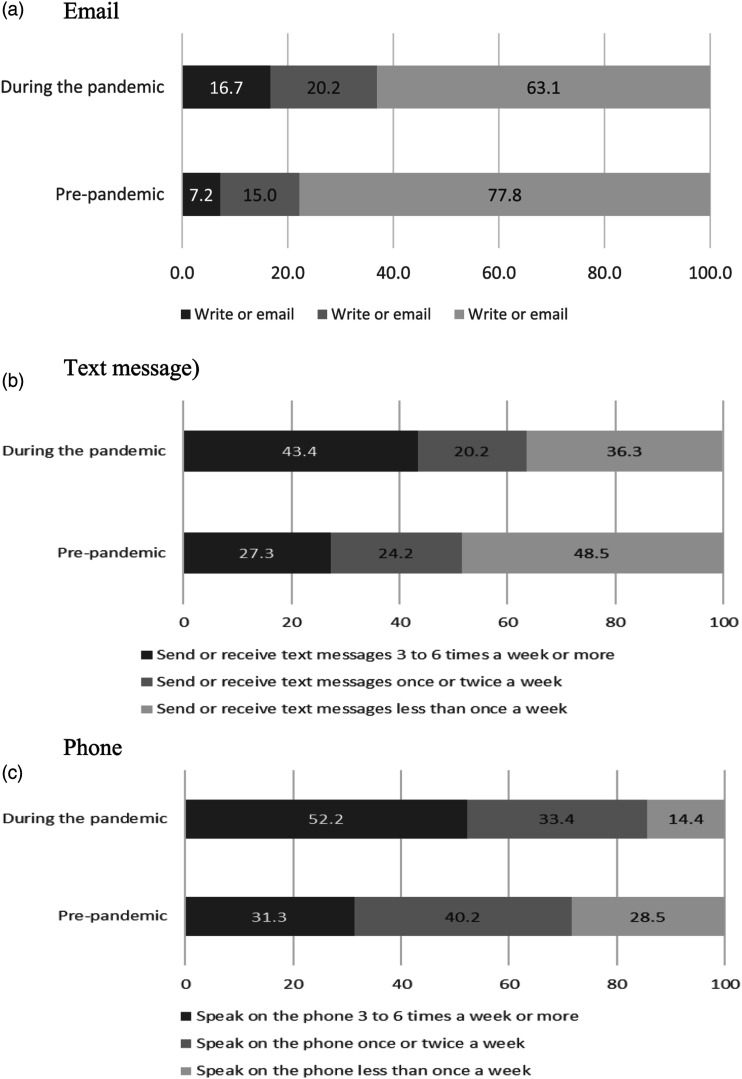
% Intergenerational contact mode and frequency pre-pandemic and during the pandemic. Source: Authors’ own analysis of the ELSA Wave 9 (2018–19) and COVID-19 sub-study (June/July 2020).

Overall, the proportion of respondents who had more frequent contact with their families increased during the pandemic compared with that pre-pandemic (Figure 1 and Supplementary Table 3). As anticipated, many older persons turned to technology contact during the pandemic, including some of those who had no frequent contact or only non-digital contact pre-pandemic. Overall, just over a quarter (25.1%) of respondents shifted towards frequent intergenerational digital communication, compared with 4% who shifted away (Table 1).

Table 3 presents the results of the multivariate models. This highlights that older cohorts had a higher likelihood of experiencing either change than younger cohorts, with the higher upward shift reflecting in part their lower use of digital communications at baseline. Women had a lower likelihood of shifting toward and shifting away from frequent intergenerational digital communication than men. Interestingly, respondents reporting two or more ADL difficulties had a higher likelihood of shifting towards frequent intergenerational digital communication than those without ADL difficulties. Older people living with someone or those with close family members also showed a lower likelihood of shifting towards frequent intergenerational digital communication than their counterparts. Internet non-users had a lower likelihood of shifting towards frequent intergenerational communication than internet users.

**Table 3.** Logistic Regression Models: Shift Towards and Shift Away From Frequent Intergenerational Digital Communication During the Pandemic.

	Shift towards frequent intergenerational digital communication b (SE)	Shift away from frequent intergenerational digital communication b (SE)
*Birth cohort*		
1946–55 (ref)		
1939–45	0.18* (0.09)	0.59** (0.21)
1930–38	0.22* (0.11)	0.85*** (0.23)
*Gender*		
Men (ref)		
Women	−0.45*** (0.08)	−0.61** (0.18)
*Education*		
Lower than O-level (ref)		
O-level	−0.05 (0.10)	−0.06 (0.23)
A-level and above	0.04 (0.10)	−0.34 (0.23)
*Wealth quintile*		
The lowest (ref)		
The second	−0.01 (0.14)	−0.30 (0.31)
The third	−0.05 (0.14)	−0.42 (0.30)
The fourth	0.15 (0.14)	−0.10 (0.29)
The highest	0.01 (0.14)	−0.47 (0.32)
*Long-standing illness*		
No (ref)		
Yes	0.11 (0.08)	0.14 (0.20)
*Number of ADL difficulties*		
0 (ref)		
1	0.03 (0.14)	0.47 (0.27)^ǂ^
2+	0.48** (0.17)	0.46 (0.35)
*Number of IADL difficulties*		
0 (ref)		
1	0.13 (0.13)	−0.03 (0.29)
2+	−0.33^ǂ^ (0.17)	0.16 (0.34)
*Living arrangements*		
Alone (ref)		
With someone	−0.21* (0.09)	0.29 (0.22)
*Has at least one close family member*		
No (ref)		
Yes	−0.85*** (0.14)	0.37 (0.43)
*Pre-pandemic internet use*		
Ever use (ref)		
Never use	−0.25* (0.11)	0.05 (0.23)
Number of observations	4180	4180
LR chi2 (17)	115.99	51.15
Prob > chi2	<0.001	<0.001
Pseudo R2	0.0246	0.0419

^ǂ^*p*<.1. *p < .05. **p < .01. ***p < .001.

Source: Authors’ own analysis of the ELSA Wave 9 (2018–19) and COVID-19 sub-study (June/July 2020).

### Have Existing Inequalities in frequent Intergenerational Digital Communication Been Amplified or Reduced During the COVID-19 Pandemic?

The net changes (shift towards subtracted by the shift away) are presented in the right-hand panel in Table 1. Women had a higher proportion of frequent intergenerational digital communication pre-pandemic than men. However, the pre-pandemic gender disparity narrowed during the pandemic as more older men shifted towards more frequent digital communication. Interestingly, a much higher proportion of older adults who reported no close family members shifted towards digital communication during the pandemic, narrowing the digital disparity in terms of family closeness. In contrast, pre-pandemic gaps between internet users and non-users in intergenerational digital communication widened during the pandemic.

We further explored whether frequent intergenerational digital communication during the pandemic was linked to health and social care usage and loneliness during the pandemic. Results show that older people with frequent intergenerational digital communication had a lower level of unmet health and social care needs (e.g. hospital operation or treatment cancelled or not accessing needed community health and social care services) and less loneliness (e.g. feeling lonely, left out, or isolated from others) than their counterparts (Supplementary Table 4).

## Discussion

Our findings show that slightly over half of older adults aged 65 and over had frequent digital contact with their families prior to the pandemic. Intergenerational digital communication was more prevalent among individuals with a higher socio-economic status and access to technology. During the pandemic, many older persons shifted towards frequent intergenerational digital contact and such shifts were observed across all sub-groups with different demographic, socio-economic characteristics and internet skills. The plausible explanation is that digital communication has switched from being an amenity to a necessity during the public health crisis as such modes of communication have become one of the only remaining vectors for social interactions (Beaunoyer et al., 2020). Ironically, the pandemic has meant that everyone has something further in common to talk about, leading to reconnections and new forms of communication across generations, such as family Zoom meetings, wedding photos shared on WhatsApp and birthday celebrations on FaceBook. An increase in digital communication has also been found in other studies (Ipsos, 2020).

Family relationships are often assumed to be a shelter from the global pandemic (Blake et al., 2020). Our research highlights that new adopters of frequent intergenerational digital contact during the pandemic are more likely to be older cohorts, men, those who experience two or more ADL difficulties, live alone and who have no close family members, leading to a narrowing of pre-pandemic disparities along these dimensions. A previous study found that a perceived lack of need is the most common reason for a household not being digitally engaged (ONS, 2019). The COVID-19 pandemic and the physical distancing measures have triggered the need for family contact at a distance. The results indicate that, in general, older adults are not averse to using digital communication technology. However, pre-pandemic gaps between internet users and non-users in intergenerational digital communication were found to have widened during the early stages of the pandemic in the USA, reflecting that those individuals who were privileged in internet experiences were more active in communicating across digital channels than their counterparts during physical distancing (Nguyen et al., 2021).

Notwithstanding the connecting role of technology in many respects, our results also highlight that a small group of older adults shifted *away* from frequent intergenerational digital contact during the pandemic. Older cohorts and men had a higher likelihood of experiencing such a shift. Potential explanations for why some people decreased their digital communication during the pandemic, rather than using it with the same or higher frequency as before, could include the loss of in-person digital support and admission to places of free internet access such as public libraries due to lockdown measures. Family and peers are key sources of digital support (Freeman et al., 2020), both of which are important for internet adoption and its continued use. Individuals who are dependent on in-person digital help from their networks might thus experience more difficulties keeping up with digital communication when such support sources are less accessible during the pandemic.

Several potential implications for a policy promoting social connections of older adults and for crisis responses emerge from our findings. Our results reflect a general increase in intergenerational digital communication during the pandemic. This provides strong support for the notion that older adults will make greater use of Information and Communication Technology (ICT) over the coming years, supporting them in performing everyday functions or in maintaining or expanding social networks (Damant & Knapp, 2015). As a result of improved communication technology, many older persons experienced enhanced associational solidarity with their families during the crisis of the COVID-19 pandemic. Thus, ITC played a critical role in meeting older people’s health and social care needs and mitigating the negative impact on well-being. However, our work also confirms concerns about the continuing digital exclusion of some older populations during the pandemic. This finding needs to be contextualised in the UK government’s Digital Inclusion Strategy, which notes that over 53% of persons who lack basic digital skills are aged over 65, and 69% are over 55 (Government Digital Service, 2014). Education programmes for digital skills may need to highlight the benefits of being online to overcome any apprehension about engagement (Ramos Garcia et al., 2021). The design of digital communication technologies will also need to continue to respond to older adults' needs and capabilities.

The COVID-19 pandemic has adversely affected the world and influenced everyone’s life. Although the results of this study reflect the context in England, a developed nation with high ICT access and usage, the growing capabilities of technology in other developed and developing countries enable increased support for communication that spans distances. Thus, these patterns may apply outside England, where there is limited face-to-face contact, and family members turn to digital forms of communication to strengthen social integration between generations. From the theoretical perspective, our results reflect that digital communications complement in-person and telephone contact. It adds new dimensions of intergenerational solidarity (e.g. associational solidarity) between generations. Future studies applying the intergenerational solidarity theoretical framework should consider such a dimension.

Our study has some potential caveats. First, we only used data from two waves of survey and were unable to capture the long-term implications of the COVID-19 pandemic on changing digital communication. Future research will need to examine the lasting effects of such changing digital communication patterns for digital inequalities. Second, we focussed on the role of communication technology in associational solidarity. We acknowledge that digital solidarity is also an important dimension of functional solidarity. However, our data do not provide details on the type of content of the digital communication exchanged between older adults and their family members necessary to study this dimension of solidarity. Future studies could examine the extent to which people utilise ICT to sustain themselves physically, emotionally and financially. Lastly, loss of follow-up might compromise the validity of outputs when the dropout rates are different between digital technology users and non-users or the participants who drop out are different from those who do not drop out. In each situation, those lost to follow-up may have a different contact willingness and/or access to ICT during the pandemic than those who completed the study. In either case, bias could affect the validity of the inferences drawn from the study.

## Supplemental Material

Supplemental material - Has COVID-19 Been the Great Leveler? The Changing Use of Intergenerational Digital Communications Amongst Older People in England During the Pandemic Click here for additional data file. Supplemental material for Has COVID-19 Been the Great Leveler? The Changing Use of Intergenerational Digital Communications Amongst Older People in England During the Pandemic by Athina Vlachantoni, Jane Falkingham, Maria Evandrou, and Min Qin in Journal of Applied Gerontology

## Note

1. ‘O-level’ was the qualification taken by this cohort at age 16 and proximately equivalent to a completion of secondary school certificate. ‘A’ levels are the qualification taken at age 18 and are required for University entrance.
