# Supplementary material for: Has COVID-19 Been the Great Leveler? The Changing Use of Intergenerational Digital Communications Amongst Older People in England During the Pandemic
Source: J Appl Gerontol. 2023 Jan 20;42(7):1517–29. doi: 10.1177/07334648231153385 (PMC9892818; doi:10.1177/07334648231153385)
Supplement: Supplemental material - Has COVID-19 Been the Great Leveler? The Changing Use of Intergenerational Digital Communications Amongst Older People in England During the Pandemic [file sj-pdf-1-jag-10.1177_07334648231153385.pdf]

Supplementary Table 1. Follow-up and lost to follow-up sample characteristics (%)

	Follow-up sample (%)	Lost follow-up (%)	P value
Total	82.8 (n=4180)	17.2 (n=868)	
Mean Age (SD)	72.3 (6.3)	76.9 (7.3)	<0.001
Gender			0.992
Men	82.8	17.2	
Women	82.8	17.2	
Birth cohort			<0.001
1946-55	89.8	10.2	
1939-45	81.2	18.8	
1930-38	63.1	36.9	
Education			<0.001
Lower than O-level	72.0	28.0	
O-level	86.5	13.5	
A-level and above	88.8	11.2	
Wealth quintile			<0.001
The lowest	70.9	29.1	
The second	74.5	25.5	
The third	83.1	16.9	
The fourth	87.4	12.6	
The highest	88.7	11.3	
Long-standing illness			<0.001
No	87.0	13.0	
Yes	80.0	20.0	
Number of ADL difficulties			<0.001
0	85.6	14.4	
1	74.8	25.2	
2+	64.1	35.9	
Number of IADL difficulties			<0.001
0	86.6	13.4	
1	76.2	23.8	
2+	60.1	39.9	
Living arrangements			<0.001
Living alone	76.3	23.7	
Living with someone	85.2	14.8	
Has at least one close family member			0.371
No	80.9	19.1	
Yes	82.9	17.1	
Pre-pandemic internet use			<0.001
Never use	62.2	37.8	
Ever use	88.9	11.1	
Pre-pandemic family contact mode and frequency			<0.001
No frequent contact either non-digital or digital	82.3	17.7	
Frequent non-digital only	72.0	28.0	
Frequent digital with or without non-digital	88.1	11.9	

P values were from Chi-squared tests for categorical variables and ANOVA test for continuous variables. Percentage and number of respondents were non-weighted.

Source: Authors' own analysis of the ELSA Wave 9 (2018-19) and COVID-19 sub-study (June/July 2020).

Supplementary Table 2. Distribution of the number of ADL and IADL difficulties (%) (n=4,180)

	Sample %	n
Number of ADL difficulties		
0	81.8	3558
1	9.8	356
2	4.1	127
3	1.7	56
4	1.4	47
5	0.9	26
6	0.3	10
Number of IADL difficulties		
0	79.3	3466
1	10.7	407
2	5.0	166
3	2.8	84
4	1.0	25
5	0.7	17
6	0.1	5
7	0.2	6
8	0.1	4

Percentage was weighted by longitudinal weight (COV19LWGT). Number of respondents was non-weighted.
Source: Authors’ own analysis of the ELSA Wave 9 (2018-19) and COVID-19 sub-study (June/July 2020).

Supplementary Table 3. Contact mode and frequency with families living outside the household pre-pandemic and during the pandemic (n=4,180).

	Pre-pandemic	During the pandemic
Meet up face to face		
-3 to 6 times a week or more	18.4	NA
-once or twice a week	34.7	NA
-less than once a week	46.9	NA
Speak on the phone		
-3 to 6 times a week or more	33.5	53.8
-once or twice a week	39.5	31.9
-less than once a week	26.9	14.2
Video-calling (e.g. Skype, FaceTime, etc)		
-3 to 6 times a week or more	NA	15.5
-once or twice a week	NA	25.2
-less than once a week	NA	59.2
Write or email		
-3 to 6 times a week or more	7.2	16.7
-once or twice a week	15.0	20.2
-less than once a week	77.8	63.1
Send or receive text messages		
-3 to 6 times a week or more	27.3	43.4
-once or twice a week	24.2	20.2
-less than once a week	48.5	36.3
Family contact mode and frequency		
-No frequent contact either non-digital or digital	19.5	7.0
-Frequent non-digital only	26.1	17.5
-Frequent digital with or without non-digital	54.4	75.4

% was weighted by longitudinal weight (COV19LWGT). NA: information not available.

Source: Authors' own analysis of the ELSA Wave 9 (2018-19) and COVID-19 sub-study (June/July 2020).

Supplementary Table 4. Frequent intergenerational digital communication, health and social care usage, and loneliness during the pandemic.

	Frequent intergenerational digital communication	Others	Total	P-value
Hospital operation or treatment canceled since coronavirus outbreak				0.036
-No	80.7	76.9	79.8	
-Yes	19.3	23.1	20.2	
Community health and social care services usage since coronavirus outbreak				<0.001
-No needs or need/attempt to contact and accessed	83.0	77.1	81.6	
-Need/attempt to contact but not accessed	17.0	22.9	18.4	
Feel lonely				0.022
-Hardly ever or never	71.9	69.7	71.3	
-Some of the time	23.8	23.3	23.7	
-Often	4.3	7.0	5.0	
Feel lack companionship				0.571
-Hardly ever or never	66.2	65.7	66.1	
-Some of the time	27.6	26.9	27.5	
-Often	6.2	7.3	6.5	
Feel left out				0.008
-Hardly ever or never	70.9	69.5	70.6	
-Some of the time	25.8	24.6	25.5	
-Often	3.2	6.0	3.9	
Feel isolated from others				0.011
-Hardly ever or never	63.4	63.9	63.5	
-Some of the time	30.1	26.5	29.2	
-Often	6.5	9.6	7.3	

Source: Authors' own analysis of the ELSA Wave 9 (2018-19) and COVID-19 sub-study (June/July 2020).

Supplementary Text

Measures: Independent variables

Demographic factors

Birth cohorts were defined on the basis of socio-historical events (Bloomberg et al., 2021), as follows: the Depression-era cohort (birth year 1930–1938), the World War 2 cohort (1939–1945), and the post-War cohort (1946–1955). Gender was a categorical variable, coded 0 for males and 1 for females.

Socio-economic factors

One measure of socio-economic status was quantified using the respondents' highest education qualification attained. This was coded as follows: 0 for less than O-level or equivalent[1], 1 for O-level or equivalent, and 2 for A-level or higher. A second variable capturing the 'permanent income' of respondents was household wealth. This was measured using quintiles of the total net non-pension household wealth, a summary measure of the value of financial, physical and housing wealth owned by the household minus any debt. The household wealth quintile was coded 0 for the lowest quintile and 4 for the highest. The data depositors generated the estimation of this variable.

Health factors

A binary categorical variable was created to measure the long-standing illness (1 for having a self-reported long-standing illness vs 0 for other categories). The survey collected information on the respondents' ability to perform various ADLs, including dressing, walking across a room, bathing, eating, getting in and out of bed, and toileting, and Instrumental Activities of Daily Living (IADLs), including using a map, **recognizing** when in physical danger, preparing a hot meal, shopping for groceries, making phone calls, taking medications,

[1] 'O-level' was the qualification taken by this cohort at age 16 and proximately equivalent to a completion of secondary school certificate. 'A' levels are the qualification taken at age 18 and are required for University entrance.

doing work around the house, and managing money. Two summary variables of reporting difficulties with ADLs and IADLs were separately derived. Reflecting the fact that the distribution of the number of ADL and IADL difficulties were skewed (Supplementary Table 2), two categorical variables were then created. The summary variable regarding the number of ADL difficulties was coded as 0=none, 1=at least one ADL difficulty, 2= two or more ADL difficulties. Similarly, the summary variable on the number of IADL difficulties was recoded as 0=none, 1=at least one IADL difficulty, 2= two or more IADL difficulties.

Familial factors

Familial factors included closeness with family members and living arrangements. The respondents' 'closeness with family member' was measured using the following survey questions:

> "How many of your children would you say you have a close relationship with? How many of these family members (brothers or sisters, parents, or grandchildren) would you say you have a close relationship with?"

A binary categorical variable was created reflecting the respondent having at least one close family member (child or immediate families) (1 for yes vs 0 for other categories). Living arrangements were captured using a simple a binary category variable derived from the household grid (1 for living with someone vs 0 for living alone).

Digital experiences

Digital experience was measured using the information on pre-pandemic Internet use from Wave 9 and coded as 1 if respondents never used the internet and 0 if they ever used the internet.
